# Frequency of Adverse Events of Antithyroid Drugs Administered during Pregnancy

**DOI:** 10.1155/2014/952352

**Published:** 2014-01-09

**Authors:** Ai Yoshihara, Jaeduk Yoshimura Noh, Natsuko Watanabe, Kenji Iwaku, Sakiko Kobayashi, Miho Suzuki, Hidemi Ohye, Masako Matsumoto, Yo Kunii, Koji Mukasa, Kiminori Sugino, Koichi Ito

**Affiliations:** Ito Hospital, 4-3-6 Jingumae, Shibuya-ku, Tokyo 150-8308, Japan

## Abstract

The frequency and types of adverse events after initial antithyroid drug (ATD) therapy during pregnancy have never been reported, nor has whether the frequency of adverse events is the same as among nonpregnant subjects ever been investigated. We investigated retrospectively the frequency of adverse events after initial ATD administration to previously untreated Graves' disease (GD) patients during pregnancy. We reviewed the charts of cases of 91 untreated pregnant women who came to our hospital for the first time and were newly diagnosed with GD during the period between January 1, 1999, and December 31, 2011. Thiamazole (MMI) was used to treat 40 patients and 51 patients were treated with propylthiouracil (PTU). Adverse events occurred in 5 patients (5/40; 12.5%) treated with MMI, and they consisted of cutaneous reactions in 5 patients. Adverse events occurred in five patients (5/51; 9.8%) treated with PTU, and they consisted of hepatotoxicity in two patients and cutaneous reactions in three patients. No patients experienced agranulocytosis or ANCA-related vasculitis. Comparison with the expected rate of adverse events in nonpregnant individuals showed that the frequency of adverse events in pregnant individuals was low.

## 1. Introduction

Thyrotoxicosis occurs in approximately 0.2% of pregnancies, and the most common cause of thyrotoxicosis is Graves' disease (GD) [1, 2]. GD is common in young women of childbearing age, and poorly controlled GD during pregnancy can cause serious complications in both the mother and the fetus. The differential diagnosis of GD from gestational thyrotoxicosis is supported by a typical goiter, the presence of TSH receptor antibody (TRAb), and signs of Graves' ophthalmopathy. Thiamazole (MMI) and propylthiouracil (PTU) are antithyroid drugs (ATDs) that are used to treat GD. ATDs are associated with a rather high frequency of adverse events; hepatotoxicity, cutaneous reactions, and agranulocytosis are the main adverse events [3]. Frequencies of adverse events in Japanese GD patients after initial ATD treatment have recently been reported [3, 4], and according to the reports the percentage of patients with aspartate aminotransferase (AST) and alanine aminotransferase (ALT) levels more than twice the upper limit of the reference range was 25.8–26.9% among patients treated with PTU as opposed to 6.6–9.0% among patients treated with MMI, and the percentage of patients with a cutaneous reaction (skin eruption or urticarial eruption) was 18.3–22.1% among the patients treated with PTU and 6.6–31.9% among the patients treated with MMI. There have been no reports on the frequencies and types of adverse events of initial ATD treatment during pregnancy. In this study we investigated the frequency of adverse events in untreated pregnant GD patients after initial ATD treatment during pregnancy.

## 2. Subjects and Methods

We reviewed the cases of 91 untreated pregnant women who came to our hospital for the first time and were newly diagnosed with GD during the period between January 1, 1999, and December 31, 2011. The diagnosis of GD was made on the basis of elevation of the serum free triiodothyronine (fT3) level and free thyroxine (fT4) level, suppression of the serum TSH level, a typical goiter, ophthalmopathy, and the presence of TSH receptor antibody (TRAb). Gestational transient thyrotoxicosis was differentiated and excluded by the presence of TRAb. Patients were assessed for adverse events by a careful health interview and physical examination. Patients visited our hospital every two weeks for two months after initiation of their treatment. Then, they were followed up every 1 to 2 months according to their thyroid function and symptoms. Serum thyroid hormone levels, TSH, AST, and ALT and hematological values were measured and a blood examination was performed at each outpatient visit. AST and ALT levels more than twice the upper limit of their reference ranges were considered evidence of hepatotoxicity as an adverse effect of the ATD.

## 3. Statistical Analysis

Data were statistically analyzed by using the chi-squared test or Fisher's exact test. Calculations were performed using the JMP software program, version 10.0.0 (SAS Institute Inc., Cary, NC). The results of the statistical analyses were considered significant at *P* values < 0.05.

## 4. Results

MMI was used to treat 40 patients, and 51 patients were treated with PTU. There were no significant differences between the two groups in serum fT3, fT4, serum TRAb, AST, or ALT values (data not shown). The characteristics of the 91 patients are shown in Table 1. Among 40 patients treated with MMI, 21 patients started treatment during the first trimester, 18 patients started during the second trimester, and one patient started during the third trimester. Adverse events occurred in 5 patients (5/40; 12.5%) treated with MMI, and they consisted of cutaneous reactions. Three of them started MMI during the first trimester of pregnancy and the other 2 patients started during the second trimester of pregnancy.

**Figure 1:**
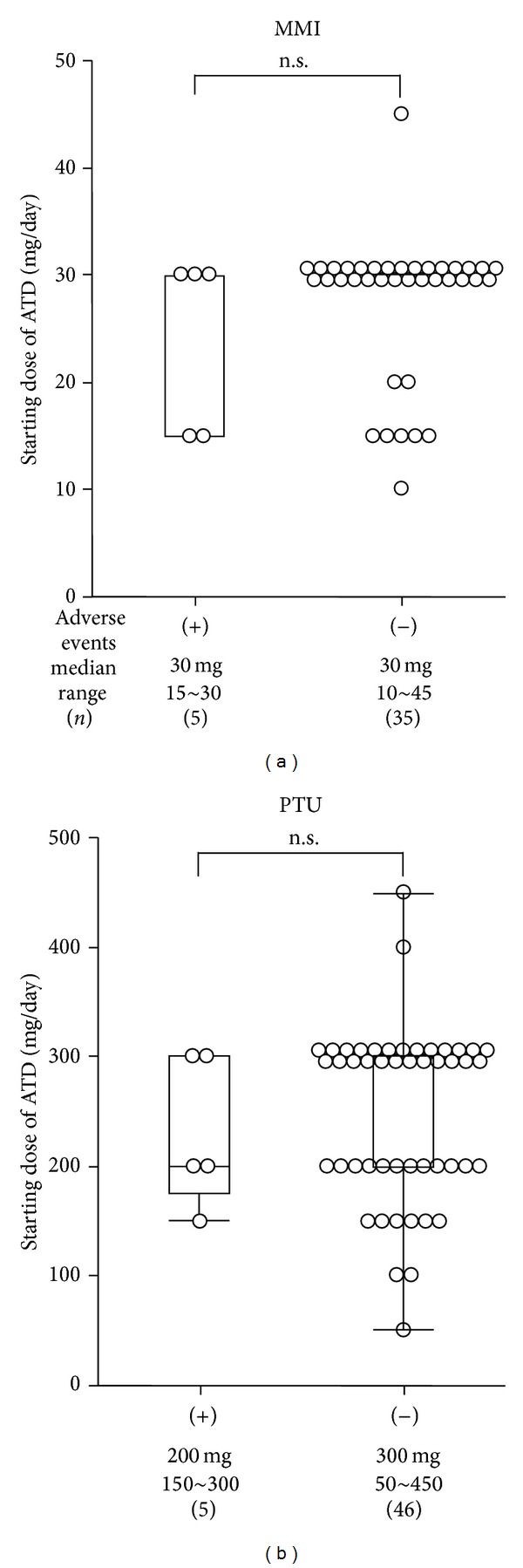
Starting dose of ATD in the group that developed adverse events and the group that did not develop adverse events.

Two of the 5 patients in the MMI group who had cutaneous reactions continued taking MMI, and their pruritus and drug rashes resolved. Treatment in the other 3 patients was switched from MMI to PTU or potassium iodide and their symptom resolved. The cutaneous reactions in these 5 patients had occurred two to six weeks after the start of MMI. All 5 patients successfully delivered full-term healthy infants. As shown in Table 1 and Figure 1, there were no significant differences between maternal age and the starting dose of MMI in the group that developed adverse events and the group that did not develop adverse events. Figure 1 shows the starting dose of MMI in the group that developed adverse events and the group that did not develop adverse events. The rate of preterm delivery was 7.5% (3/40), and one newborn had a congenital abnormality, an omphalocele. The mother of the infant with the omphalocele had started taking a 30 mg dose of MMI daily at 4 weeks of pregnancy and had continued until delivery.

Among 51 patients treated with PTU, 26 patients started treatment during the first trimester, 22 patients started during the second trimester, and 3 patients started during the third trimester. Adverse events occurred in five patients (5/51; 9.8%); four of them started PTU during the first trimester of pregnancy and the other one patient started during the second trimester of pregnancy. Adverse events consisted of hepatotoxicity in two patients and cutaneous reactions in three patients. No patients experienced hematologic side-effects including agranulocytosis [5] or ANCA-related vasculitis [6]. The two patients who developed hepatotoxicity discontinued PTU. In one of the patients, PTU was started from the fourth week of pregnancy and the AST and ALT levels had increased to more than twice the upper limit of the normal range at 9 weeks after the start of treatment. In the other patient, PTU was started from the fourth week of pregnancy and the AST level had increased to 3 times the upper limit of the normal range and the ALT level to more than 5 times the upper limit of the normal range at two weeks, and PTU was discontinued. Both patients' liver function improved immediately after PTU was discontinued. Treatment in these two patients was switched from PTU to potassium iodide, and their thyroid status was well controlled. Both of them successfully delivered full-term healthy infants. Cutaneous reactions occurred in three patients treated with PTU. Two of them continued taking PTU, and their pruritus and drug rashes resolved. It was necessary to discontinue PTU in the third patient. Her treatment was switched from PTU to potassium iodide, and she successfully delivered a full-term healthy infant. The cutaneous reactions in these three patients developed two to ten weeks after the start of PTU treatment. As shown in Table 1 and Figure 1, there were no significant differences between maternal age and the starting dose of PTU in the group that developed adverse events and the group that did not develop adverse events. The rate of preterm delivery was 7.8% (4/51), and none of the infants of the mothers treated with PTU were born with a congenital abnormality. There was no significant difference in frequency of adverse events between MMI and PTU.

## 5. Discussion

Hyperthyroidism in pregnancy is a serious condition that entails an increased risk of adverse obstetric outcomes, including miscarriage, stillbirth, preterm birth, and intrauterine growth restriction. ATDs should be used to treat hyperthyroidism due to GD during pregnancy. In the last decades, MMI and PTU have been used equally to treat GD during pregnancy, regardless of trimester. In recent years, PTU has been the preferred drug because of concerns about teratogenicity associated with MMI during the first trimester of pregnancy. The ATA recommendation suggests using PTU during the first trimester and MMI during the second trimester. The majority of cases in our study were treated before the recommendation was published, and in 21 patients treatment with MMI was started in the first trimester. The only infant born with an omphalocele was delivered by a mother treated with MMI starting in the first trimester, and this congenital abnormality may be related to administration of MMI starting in the first trimester of pregnancy [7]. The rate of preterm delivery by patients treated with an ATD was high in comparison with the rate of 5% in the general population [8]. Before treating patients with an ATD they should be informed of the possible development of adverse events such as pruritic rashes, liver dysfunction, and agranulocytosis. In Japan, a physical examination and blood testing are mandated every two weeks especially within 2 months after the start of ATD treatment to enable early detection of agranulocytosis and liver dysfunction. This practice may be useful for early detection of mild cutaneous reactions and liver dysfunction associated with ATD treatment.

A previous study of the frequency of adverse events of MMI and PTU in nonpregnant GD patients at our hospital reported the development of cutaneous reactions in 31.9% of the patients treated with MMI and in 18.3% of the patients treated with PTU. The frequency of hepatotoxicity was 9.0% in the MMI group and 25.8% in the PTU group. Our study in pregnant GD patients showed that the frequency of cutaneous reactions among the patients treated with MMI was 12.5% and 5.9% among the patients treated with PTU. No patients treated with MMI developed hepatotoxicity, and the frequency of hepatotoxicity among the patients treated with PTU was 3.9%. Since the study design was completely different, it is difficult to compare the frequencies of adverse events between the pregnant patients and the nonpregnant patients. Comparison with the expected rate of adverse events in nonpregnant individuals showed that the frequency of adverse events in pregnant individuals was low. At least, the frequencies of adverse events in the pregnant patients were not higher than in the nonpregnant patients. The reason for the lower frequencies of adverse drug reactions in the pregnant women is unclear. Reduced immune reaction during pregnancy may be the cause of low frequency of adverse reaction. However, whether the mechanisms of the rashes or hepatotoxicity induced by ATDs are related to immune reactions has never been investigated. It should be noted that the occurrence of severe liver injury in patients treated with PTU is rare. It has been estimated that 4 of the approximately 4000 pregnant women treated with PTU each year in the United States develop PTU-related severe liver injury [9]. Six cases of PTU-induced hepatitis during pregnancy have been reported in the medical literature [10–13]. Based on this small sample the outcome appears to be particularly poor in pregnancy, because there was 1 maternal death and 2 patients required a liver transplant. One case of neonatal hepatitis as an adverse effect of maternal treatment with PTU has also been reported [14].

Limitation of this study was that the number of subjects was relatively small because we selected untreated hyperthyroid pregnant women diagnosed with GD for the first time during their pregnancy. Prospective study is preferable; however, it seems to be difficult because pregnant women newly diagnosed as GD during pregnancy are rare, and initial MMI administration to untreated GD patients during the first trimester of pregnancy is not recommended from guideline of Endocrine Society and guideline of the American Thyroid Association [15, 16].

In conclusion, this is the first study that studied the frequencies of adverse events after initial treatment with an ATD during pregnancy in newly diagnosed GD patients.

## Figures and Tables

**Table 1:** The ages and initial dose of the patients treated with antithyroid drug who developed and did not develop adverse events.

			Age, yr		Initial dose (mg/day)			
	No. of patients		Mean ± SD		Median		Range	
	MMI	PTU	MMI	PTU	MMI	PTU	MMI	PTU
Without adverse events	35	46	29.7 ± 4.9	32.1 ± 4.7	30	300	10~45	50~450
With adverse events	5	5	29.2 ± 2.7	33.3 ± 4.2	30	200	15~30	150~300

*P* value			n.s.	n.s.	n.s.	n.s.		

There were no significant differences between maternal age and the starting dose of ATD (MMI and PTU) in the group that developed adverse events and the group that did not develop adverse events.

There was no significant difference in frequency of adverse events between MMI and PTU.
